# The *Misleading* count: an identity-based intervention to counter partisan misinformation sharing

**DOI:** 10.1098/rstb.2023.0040

**Published:** 2024-03-11

**Authors:** Clara Pretus, Ali M. Javeed, Diána Hughes, Kobi Hackenburg, Manos Tsakiris, Oscar Vilarroya, Jay J. Van Bavel

**Affiliations:** ^1^ Department of Psychobiology and Methodology of Health Sciences, Universitat Autònoma de Barcelona, 08193 Barcelona, Spain; ^2^ Department of Psychiatry and Forensic Medicine, Universitat Autònoma de Barcelona, 08193 Barcelona, Spain; ^3^ Center of Conflict Studies and Field Research, ARTIS International, St Michaels, MD 21663, USA; ^4^ Department of Psychology and Center for Neural Science, New York University, New York, NY 10003, USA; ^5^ Centre for the Politics of Feelings, School of Advanced Study, Royal Holloway, University of London, London WC1E 7HU, UK; ^6^ Department of Psychology, Royal Holloway, University of London, Egham, Surrey TW20 0EX, UK

**Keywords:** misinformation, social media, social norms, social identity, intervention

## Abstract

Interventions to counter misinformation are often less effective for polarizing content on social media platforms. We sought to overcome this limitation by testing an identity-based intervention, which aims to promote accuracy by incorporating normative cues directly into the social media user interface. Across three pre-registered experiments in the US (*N* = 1709) and UK (*N* = 804), we found that crowdsourcing accuracy judgements by adding a *Misleading* count (next to the *Like* count) reduced participants' reported likelihood to share inaccurate information about partisan issues by 25% (compared with a control condition). The *Misleading* count was also more effective when it reflected in-group norms (from fellow Democrats/Republicans) compared with the norms of general users, though this effect was absent in a less politically polarized context (UK). Moreover, the normative intervention was roughly five times as effective as another popular misinformation intervention (i.e. the accuracy nudge reduced sharing misinformation by 5%). Extreme partisanship did not undermine the effectiveness of the intervention. Our results suggest that identity-based interventions based on the science of social norms can be more effective than identity-neutral alternatives to counter partisan misinformation in politically polarized contexts (e.g. the US).

This article is part of the theme issue ‘Social norm change: drivers and consequences’.

## 1. Introduction

Online misinformation poses a substantial threat to democracy and public health. From the *infodemic* surrounding the COVID-19 pandemic [1,2] to the election fraud disinformation campaign that led to the 6 January assault on the US Capitol [3], misinformation appears to be a significant risk to public health and democratic institutions. On Twitter, falsehoods spread significantly farther, faster and deeper than true stories–and this was especially true for political and emotional stories [4]. Online misinformation drives user engagement [4–8], capturing 2.3% of Web traffic and 14% of Facebook engagement according to recent estimates. As such, social media companies have few incentives to eliminate misinformation. Existing infrastructure for online content moderation has also proven unable to meet rapidly increasing demand: moderation is often outsourced to foreign workers who need to make split-second decisions on content that is highly dependent on local social and political contexts. Therefore it is critical to create systemic changes to social media infrastructure that can effectively reduce misinformation sharing in a way that is scalable, context-sensitive and effective among at-risk groups. In the current paper we develop and evaluate an identity-based intervention for reducing online misinformation sharing and compare it with popular approaches to reduce misinformation.

One of the most popular moderation-free approaches to combating misinformation is ‘accuracy nudging’: presenting users with visual or textual cues that remind them to be accurate. This approach is based on the idea that people largely share misinformation because they are inattentive or lack analytical thinking skills [9] and has received extensive empirical investigation [10]. However, recent studies and a meta-analysis suggest that the effect of accuracy nudges may be relatively weak [11], especially among conservatives, Republicans, and far-right supporters [12,13]. Similarly, a recent meta-analysis found that most strategies for debunking misinformation were not very effective overall (Cohen's *d* = 0.19), and were even less effective when the issue was politically polarized [14]. As such, there is an urgent need to develop effective and scalable correction strategies for misinformation in the political domain that works across the political spectrum.

According to the Identity-Based Model of Political Belief [15], individuals are more likely to believe and share misinformation when their partisan motives outweigh accuracy concerns [16,17]. This helps explain why partisan misinformation may be more difficult to debunk—especially in polarized contexts. For instance, we recently found that partisans who were highly devoted to a political party were more likely to spread misinformation than centrist votes and were unresponsive to fact-checking [13]. The fact that interventions to counter misinformation based on analytical thinking are relatively ineffective for political extremists and right-wing users is practically important since these populations contribute the most to the spread of misinformation, at least in the US [18–20]. It also underscores the need to incorporate social identity and group norms in the design of misinformation interventions.

Online misinformation is usually embedded in an interactive social environment (i.e. social media platforms) with visible social engagement metrics (e.g. number of *Like*s), which have been found to increase people's vulnerability to misinformation [21]. However, this also offers great potential for interventions based on social psychology [22]. For instance, actual reporting of fake news [23] and willingness to correct misinformation online [24] have been associated with social norms (i.e. beliefs on what others do or deem desirable, see [25]) about these behaviors. The effect of social norms may be more complex when it comes to misinformation about polarizing issues (e.g. election fraud allegations) since beliefs and behaviour are likely to be determined by the intergroup and intragroup dynamics of fellow partisans. In line with Social Identity Theory [26] and Self-Categorization Theory [27], opinions from the in-group tend to induce greater conformity than opinions from the out-group [28]. Thus, in a polarized digital environment, people may be more likely to conform to in-group social norms than to social norms by general users.

Here, we propose that exposing individuals to normative accuracy judgements by their in-group (versus general others) may be helpful to counter partisan misinformation (e.g. misinformation that favours specific in-group partisan stances). Indeed, laypeople are relatively good at distinguishing low-quality news content [29], raising the possibility of crowdsourcing accuracy judgements. This norms-based approach could be particularly useful for misinformation on politically polarizing issues (e.g. attitudes towards immigration and universal healthcare, see [30,31]), which people are more likely to share than misinformation on non-polarizing issues (e.g. infrastructure, see [13]). Crowdsourcing only from the in-group may also contribute to correcting inaccurate perceptions of in-group norms over particular issues, which could help reduce misperceived polarization [32]. Therefore, identity-based interventions that leverage normative cues to nudge people into being more accurate may be an effective and scalable approach to moderating online misinformation.

### (a) Current research

In the present work, we tested the effect of normative accuracy judgements from the in-group to reduce sharing of partisan misinformation in three pre-registered online experiments with Democrats and Republicans in the US (*N* = 1709) and Labour and Conservative voters in the UK (*N* = 804). Although both contexts are politically polarized, a cross-country analysis found higher levels of affective polarization in the US compared with the UK [33]. We asked participants how likely they would be to share a series of simulated social media posts composed by different in-group political leaders (e.g. Bernie Sanders for Democrats) that contained inaccurate information relevant to politically polarizing issues (e.g. immigration, homelessness). The intervention consisted of adding a *Misleading* count next to the *Like* count. Half of the participants were told the *Misleading* count reflected in-group norms, i.e. the number of fellow Democrats/Republicans who had tagged the post as misleading (identity-relevant condition). The other half were told the *Misleading* count reflected the norms of general users (identity-neutral condition). We compared this intervention with widely used interventions to counter misinformation, including (a) the official Twitter tag, a warning that precludes social media users from further sharing the posts ('This Tweet can't be replied to, shared or liked'), and (b) an accuracy nudge adapted from Pennycook *et al.* [34] ('To the best of your knowledge is the above statement accurate?'). This allowed us to test the relative efficacy of different popular interventions against the identity-based intervention.

We predicted that people would report a lower likelihood of sharing social media posts in response to seeing the *Misleading* count compared with no count (H1). In Studies 2 and 3, we also expected the *Misleading* count to be more effective in reducing sharing whenever the count was 80 compared with 20% of the *Like* count (H2). Since politically polarizing issues typically involve absolutist stances over moral issues, which are particularly resistant to trade-offs and social influence [35], we expected a reduced effect of the *Misleading* count when the posts were relevant to polarizing (versus non-polarizing) issues in Experiment 1 (H3 in Experiment 1). We also expected the *Misleading* count to be similarly effective in reducing sharing of social media posts among Democrats and Republicans in the US, and among Labour and Conservative voters in the UK (H3 in Experiments 2 and 3). Finally, because people are responsive to in-group norms specifically [36], we expected the *Misleading* count to be more effective when it reflected in-group norms compared with general users' norms (H4).

## 2. Material and methods

The data and code employed in the analyses are available at https://osf.io/dmxbt/. The pre-registrations for the three studies can be found at https://osf.io/xng3h (Experiment 1), https://osf.io/nmwvs (Experiment 2) and https://osf.io/m9hg3 (Experiment 3).

### (a) Participants

We recruited 401 Democratic and 402 Republican voters in the US for Experiment 1, 402 Labour voters and 402 Conservative voters in the UK for Experiment 2, and 453 Democratic and 452 Republican voters in the US for Experiment 3 by means of an online panel (Prolific). Inclusion criteria included being 18 or older and having voted for the relevant political party in the two previous presidential elections (see demographic information in electronic supplementary material, table S1 and power analysis in electronic supplementary material, methods, Participants).

### (b) Material

The posts were designed to look like Tweets and contained inaccurate information about a series of political issues that we expected would be engaging to participants (see electronic supplementary material, tables S2–S4). The use of artificial rather than real misinformation allowed us to control both content and grammatical structure. The posts were tested for perceived accuracy, salience, familiarity and importance in a series of pilot studies with independent samples matched for country of residence and political affiliation (see electronic supplementary material, Methods, Materials and table S5). The pilot studies also confirmed that the information contained in the posts was neither too plausible nor too implausible to avoid ceiling and floor effects in participants’ likelihood of sharing (see electronic supplementary material, table S5). Participants were exposed to information aligned with their political affiliation (e.g. in favour of universal healthcare for Democrats).

In Experiment 1, half of the social media posts included information about politically polarizing issues (e.g. immigration, universal healthcare), and the other half included non-polarizing issues (e.g. infrastructure). In Experiments 2 and 3, all the social media posts conveyed information about polarizing issues (e.g. immigration, universal healthcare). As expected, a larger proportion of participants held absolutist stances (resistant to economic trade-offs) over issues that we proposed as polarizing as compared with issues that we proposed as non-polarizing. Whether participants held absolutist stances over each issue was assessed in the same survey (see electronic supplementary material, Materials and table S6).

### (c) Procedure

We launched surveys asking Democrats and Republicans in the US (Experiments 1 and 3) and Labour and Conservative voters in the UK (Experiment 2) to rate the likelihood of sharing a series of social media posts composed by different political leaders of the party they voted for in the last elections. We tested different variations of an identity-based intervention: we included a *Misleading* count next to the *Like* count, which we told participants reflected in-group norms, i.e. the number of fellow Democrats/Republicans who had tagged the post as misleading. The *Misleading* count was always lower than the *Like* count, as expected for highly partisan content. Social media posts with and without interventions were presented in a randomized order.

**Figure 1.**
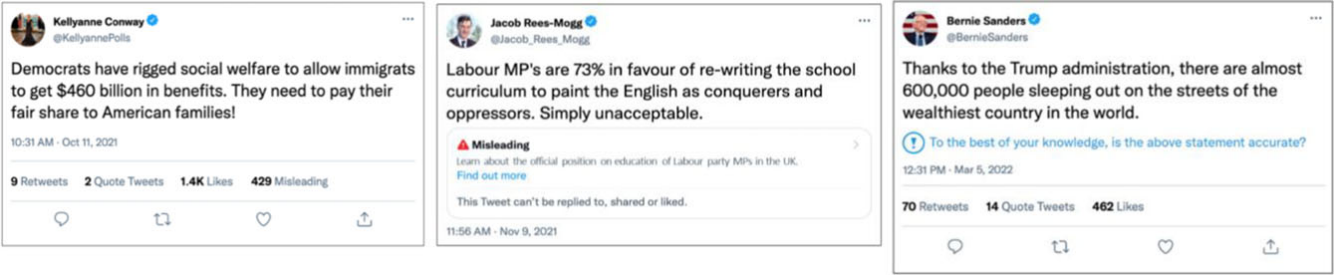
Employed interventions. Examples of the employed interventions including (*a*) the *Misleading* count condition (30% of the *Like* count) used in Experiment 1, (*b*) the official Twitter Misleading tag used in Experiment 2, and (*c*) the accuracy nudge used in Experiment 3.

In Experiment 1 (*N* = 803), half of the social media posts contained a *Misleading* count that was 30% of the *Like* count (figure 1*a*) and the other half did not contain any intervention (control condition).

In Experiment 2 (*N* = 804), 25% of the social media posts contained a low *Misleading* count (20% of the *Like* count), 25% contained a high *Misleading* count (80% of the *Like* count), 25% contained an official Twitter Misleading tag (figure 1*b*), and 25% did not include any intervention (control condition). Because the official Twitter Misleading tag prevents participants from sharing the post, we asked participants how likely they would be to share the post through other means (e.g. taking a screenshot). Since sharing messages in this condition requires extra effort, the dependent variable (sharing intentions) is psychologically different in the official Twitter tag compared with the *Misleading* count conditions. The value of including an established intervention against misinformation (the official Twitter tag) lies in the comparison of final outcomes (whether the message is likely to be shared or not, independently of the means), which is relevant for potential implementations of the *Misleading* count.

In Experiment 3 (*N* = 905), we used the same design as in Experiment 2 but instead of the official Twitter Misleading tag, we compared the high and low *Misleading* count to an accuracy nudge (‘To the best of your knowledge, is the above statement accurate?’, adapted from Pennycook *et al.* [34]). Of note, the accuracy nudge was included directly on the social media posts (figure 1*c*) unlike in the original setting, where it was administered as a separate intervention at the beginning of the experiment [34]. Thus, participants were exposed to the accuracy nudge more intensively than in the original setting. As such, the accuracy nudge effects might be stronger here than in the traditional implementation.

Across the three studies, all participants were exposed to all interventions and the control condition (within-subjects factor). All three studies included an additional between-subjects control condition so that half the sample was told that the *Misleading* count reflected general users' norms, i.e. the number of general users who had tagged the post as misleading (tagged by anyone) instead of only fellow Democrats/Republicans (tagged by in-group) (see electronic supplementary material, Methods for more details).

## 3. Results

### (a) Effect of the intervention

**Figure 2.**
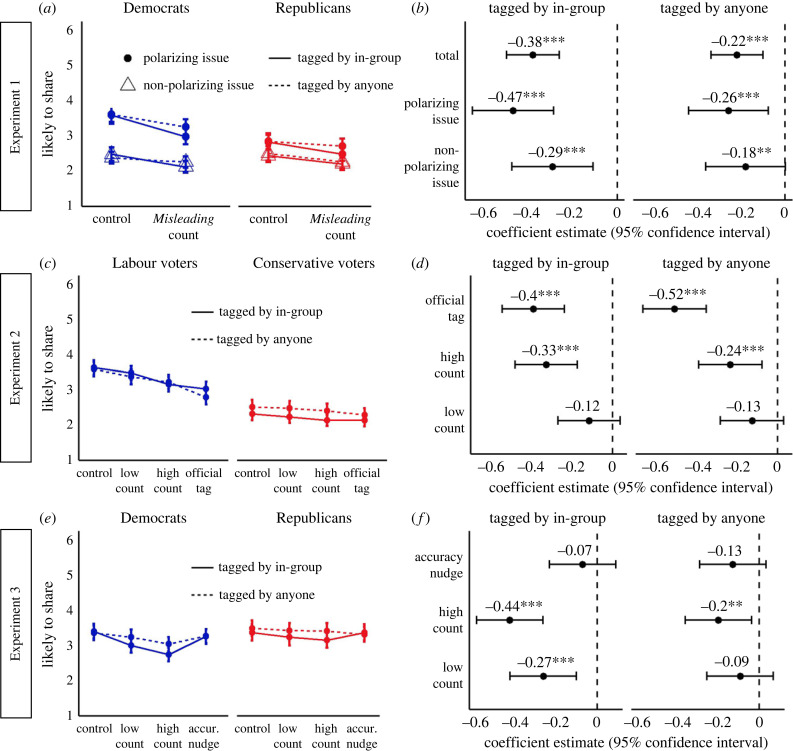
Effect of the intervention across experiments. (*a,c,e*) Likelihood of sharing social media posts on polarizing issues (Experiments 1–3) and non-polarizing issues (Experiment 1) in response to the *Misleading* count (Experiments1–3), the official Twitter tag (Experiment 2) and the accuracy nudge (Experiment 3) compared with control by group and condition (tagged by in-group’ and ‘tagged by anyone). In Experiment 1, the *Misleading* count was always 30% of the *Like* count. In Experiments 2 and 3, the *Misleading* count was presented in two conditions: high count (80% of the *Like* count) and low count (20% of the *Like* count). The absolute *Misleading* count was two orders of magnitude higher in Experiment 1 as compared with Experiments 2 and 3 (e.g. 10 000 versus 100). Error bars represent 95% confidence intervals. (*b,d,f*) Coefficient estimates of the contrast between each intervention compared wiith control for social media posts relevant to polarizing (Experiments 1–3) and non-polarizing issues (Experiment 1) by condition (tagged by in-group and tagged by anyone). Error bars represent 95% confidence intervals. Asterisks represent levels of significance. *p-value <0.05, **p-value <0.01, ***p-value <0.001.

As predicted (H1), including a *Misleading* count next to the *Like* count (figure 1*a*) reduced participants' likelihood of sharing misinformation compared with the no intervention control condition across all three studies (*p* < 0.001, Cohen's *d* = 0.20, see electronic supplementary material, table S7a, and figure 2). In addition, participants were sensitive to the proportion of Misleadings compared with the number of Likes (H2) both in the US (Experiment 2) and the UK (Experiment 3). Specifically, respondents reported a slightly lower likelihood of sharing when the *Misleading* count was 80 compared with 20% of the *Like* count in the US, *M*_diff_ = −0.14, 95% CI [−0.24, −0.04], *z*-score = −3.56, *p* = 0.002, *d* = 0.08, and in the UK, *M*_diff_ = −0.16, 95% CI [−0.28, −0.07], *z*-score = −4.43, *p* = 0.001, *d* = 0.10. In Experiment 2, the high *Misleading* count condition (80% of the *Like* count) was outperformed by the official Twitter Misleading tag, *M*_diff_ = −0.17, 95% CI [−0.26, −0.08], *z*-score = −4.61, *p* < 0.001, *d* = 0.12, which prevents Twitter users from further sharing the post (‘This Tweet can't be replied to, shared or liked’, figure 1*b*). However, in Experiment 3, the high *Misleading* count worked better than the accuracy nudge ( figure 1*c*) in reducing participants' likelihood of sharing misinformation, *M*_diff_ = −0.22, 95% CI [−0.32, −0.12], *z*-score = −5.60, *p* < 0.001, *d* = 0.13. As such, the *Misleading* count appears to be a relatively effective strategy for reducing misinformation sharing.

In an explorative analysis looking at the dichotomized response variable (likely versus not likely to share) across the three experiments, the number of participants likely to share the social media posts (likelihood > 3) was reduced by around 25% in response to the *Misleading* count versus control in the in-group condition, *M*_diff_ = −0.26, 95% CI [−0.35, −0.16], *t*_95_ = −6.85, *p* *<* 0.001, as compared with a 12% reduction in the general users’ condition versus control, *M*_diff_ = −0.12, 95% CI [−0.22, −0.03], *t*_95_ = −3.30, *p* *=* 0.007 (interaction effect: *B* = 0.13, 95% CI [0.03, 0.23], *t*_94_ = 2.51, *p* *=* 0.014). The same model revealed a 34% reduction in the number of participants likely to share the social media posts in response to the official Twitter tag versus control (*B* = −0.34, 95% CI [−0.45, −0.23], *t*_98_ = −6.13, *p* *<* 0.001) and a 5% reduction in response to the accuracy nudge versus control (*B* = −0.05, 95% CI [−0.16, 0.06], *t*_98_ = −0.91, *p* *=* 0.366).

To test the possibility of demand effects (i.e. to see if the ‘study would become quite obvious' over time), we tested if the initial effects (first four trials) were different from the overall effects in an exploratory analysis. Presumably, the effect would change over time if demand effects increasingly came into play. However, the results of this additional analysis revealed that the early effects were nearly identical to the overall effects (see electronic supplementary material, table S8). Thus, potential demand effects do not appear to have changed our results in any measurable way.

### (b) Extreme partisanship

Interventions to counter misinformation are often less effective when partisan incentives outweigh accuracy concerns—for instance, when misinformation is framed in terms of group-relevant politically polarizing issues, and for individuals who highly identify with the group [13]. Thus, we tested if the *Misleading* count was also less effective in these conditions. In Experiment 1, we compared the effect of the intervention for misinformation relevant to politically polarizing issues compared with non-polarizing issues (as measured in our surveys, see the percentage of participants with absolutist stances over each issue in electronic supplementary material, table S6). Contrary to our expectations (H3 in Experiment 1), the *Misleading* count (versus control) was actually *more* effective for social media posts on polarizing issues (e.g. immigration, homelessness) than non-polarizing issues (interaction effect: *B* = −0.13, 95% CI [−0.25, −0.002], *t*_2406_ = −2.00, *p* *=* 0.046, figure 2*a,b*).

In terms of identity fusion (i.e. visceral oneness with a group or leader, see [37]), we found no interaction effect between the intervention and identity fusion with the leader or with the political party (*p* > 0.093). If anything, there was a trend in the opposite direction in Experiment 1 (interaction effect: *B* = −0.16, 95% CI [−0.36, −0.03], *t*_801_ = −1.68, *p* *=* 0.093). Specifically, participants who reported feeling fused with the leader were more responsive to the *Misleading* count (versus control), *M*_diff_ = −0.44, 95% CI [−0.62, −0.26], *t*_801_ = −8.17, *p* *<* 0.001, *d* = 0.27, than non-fused participants, *M*_diff_ = −0.28, 95% CI [−0.35, −0.21], *t*_801_ = −8.17, *p* *<* 0.001, *d* = 0.19. Therefore, extreme partisanship measured as identity fusion both with leaders and with political parties did not undermine the effectiveness of the intervention.

### (c) Political affiliation

**Figure 3.**
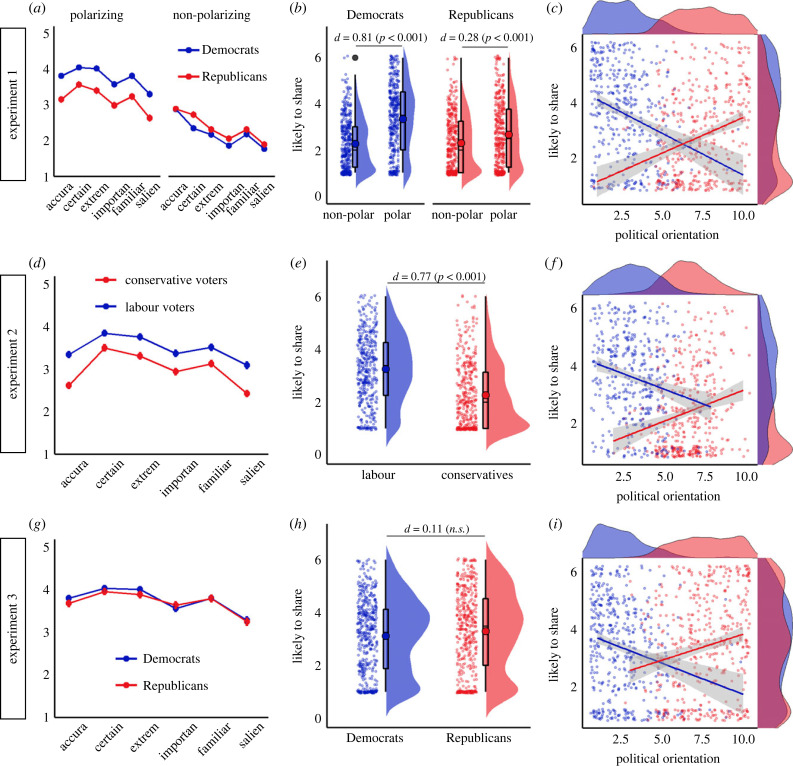
Perceived accuracy, attitude strength, and likelihood of sharing social media posts across experiments. (*a,d,g*) Perceived accuracy, attitude strength (certainty, extremity, and importance), familiarity, and salience of the used social media posts by group (Exp. 1–3) and type of issue (polarizing and non-polarizing) (Exp. 1) as tested in pilot studies (Exp 1: N = 370; Exp 2: N = 80; Exp 3: N = 234). (*b,e,g*) Likelihood of sharing social media posts on polarizing issues (Exp. 1–3) and non-polarizing issues (Exp. 1) as a function of political affiliation. (*c,f,i*) Extreme political orientation was associated with an increased likelihood of sharing social media posts in the control condition (no interventions) across samples and political groups (liberals in blue and conservatives in red). Notably, U.S. samples in Exp. 1 and 3 were more polarized in terms of political orientation compared to the UK sample in Exp. 2.

In contrast to our pre-registered hypothesis (H3 in Experiments 2 and 3), Democrats and Labour voters were generally more responsive to the intervention than Republicans and Conservatives, respectively (see electronic supplementary material, table S7c, and figure 2*a,c,e*). This effect was less clear in Experiment 1, where the *Misleading* count (versus control) was only marginally better (*p* = 0.072) at reducing the likelihood of sharing misinformation among Democrats, *M*_diff_ = −0.36, 95% CI [−0.47, −0.27], *t*_801_ = −7.88, *p* *<* 0.001, *d* = 0.23, compared with Republicans, *M*_diff_ = −0.24, 95% CI [−0.33, −0.15], *t*_801_ = −5.34, *p* *<* 0.001, *d* = 0.17. In Experiment 2, group differences were most apparent in the high *Misleading* condition, where Labour voters in the UK reduced their likelihood of sharing in response to the intervention (versus control) to a greater extent, *M*_diff_ = −0.42, 95% CI [−0.56, −0.29], *z*-score = −8.20, *p* *<* 0.001, *d* = 0.29, than Conservative voters, *M*_diff_ = −0.14, 95% CI [−0.28, −0.02], *z*-score = −2.90, *p* *=* 0.020, *d* = 0.11. However, US Republicans and UK Conservatives were overall less likely to share the social media posts that were presented to them both in Experiment 1, *M*_diff_ = −0.31, 95% CI [−0.48, −0.14], *t*_801_ = −3.58, *p* *<* 0.001, *d* = 0.21 (figure 3*a*), and in Experiment 2, *M*_diff_ = −0.98, 95% CI [−1.16, −0.81], *t*_802_ = −10.89, *p* *<* 0.001, *d* = 0.68 (figure 3*d*). Thus, it could be that the reduced effect of the intervention in these samples was due to floor effects in likelihood of sharing.

Therefore, in Experiment 3, we made sure the stimuli for Republicans were matched with those presented to Democrats in perceived accuracy, attitude strength, familiarity and salience (see electronic supplementary material, table S5, and figure 3*g*). As a result, we found only a marginal difference in overall sharing between groups in Experiment 3, *M*_diff_ = −0.18, 95% CI [−0.38, 0.01], *t*_897_ = −1.83, *p* *=* 0.067, *d* = 0.11, and if anything, the trend indicated higher sharing among Republicans (figure 3*h*). Despite having similarly appealing social media posts for Democrats and Republicans in Experiment 3, the effect of the intervention (versus control) was still larger in Democrats than Republicans (see electronic supplementary material, table S7c, and figure 2*e*), which is consistent with the accuracy nudge intervention [12].

### (d) Group norms

In line with our hypothesis (H4), the *Misleading* count was more effective when it reflected in-group norms (i.e. the number of fellow Republicans/Democrats who had tagged the post as misleading), as compared with the norms of general users (see electronic supplementary material, table S7b, and figure 2*b,d,f*). Specifically, in Experiment 1 (intervention × in-group interaction: *B* = −0.16, 95% CI [−0.29, −0.04], *t*_801_ = −2.57, *p* *=* 0.01), the *Misleading* count (versus control) was associated with a steeper reduction in the likelihood of sharing in the in-group condition, *M*_diff_ = −0.38, 95% CI [−0.47, −0.29], *t*_810_ = −8.40, *p* *<* 0.001, *d* = 0.26, compared with the general users' condition, i.e. tagged by anyone, *M*_diff_ = −0.22, 95% CI [−0.31, −0.13], *t*_801_ = −4.88, *p* *<* 0.001, *d* = 0.14. In Experiment 3, the effect of the in-group was particularly important for the low *Misleading* count condition (intervention × in-group interaction: *B* = −0.17, 95% CI [−0.34, −0.04], *t*_2694_ = −2.25, *p* *=* 0.024), such that the low *Misleading* count (20% of the Likes) was associated with reduced misinformation sharing compared with control only in the in-group condition, *M*_diff_ = −0.27, 95% CI [−0.41, −0.13], *z*-score = −4.91, *p* *<* 0.001, *d* = 0.16, but not in the general users' condition, *M*_diff_ = −0.09, 95% CI [−0.24, 0.05], *z*-score = −1.72, *p* *=* 0.312, *d* = 0.06. Similarly, the high *Misleading* count (80% of the Likes) versus control was more effective in the in-group condition, *M*_diff_ = −0.44, 95% CI [−0.58, −0.30], *z*-score = −7.97, *p* *<* 0.001, *d* = 0.26, than the general users' condition, *M*_diff_ = −0.20, 95% CI [−0.34, −0.06], *z*-score = −3.71, *p* *=* 0.001, *d* = 0.12 (intervention × in-group interaction: *B* = −0.23, 95% CI [−0.38, −0.08], *t*_2694_ = −3,01, *p* *=* 0.003).

However, the effect of the in-group was only observed in the US sample (Experiments 1 and 3) and was not found in the UK sample (Experiment 2, *p* > 0.194, see electronic supplementary material, table S7b), which was less polarized in terms of political orientation (figure 3*f*) than the US samples (figure 3*c*,*i*). Thus, incorporating the in-group dimension may be more effective in highly polarized US voters (see political orientation distribution in figure 3*c*,*i*) and less so among less polarized UK voters (see political orientation distribution in figure 3*f*).

### (e) Engagement

The social media posts used in Experiment 1 were designed to have higher social engagement metrics (from 782 to 16 900 *Misleading*s) than those in Experiments 2 and 3 (from 62 to 199 *Misleading*s*,* see all items in electronic supplementary material, tables S5–S7). In an explorative analysis, we found that participants were less likely to share misinformation the higher the absolute count of *Misleading*s was (Experiment 1: *B* = −0.00002, 95% CI [−0.00003, −0.00001], *t*_9721_ = −3.51, *p* *<* 0.001; Experiment 2: *B* = −0.0006, 95% CI [−0.0008, −0.0004], *t*_2432_ = −7.36, *p* *<* 0.001; Experiment 3: *B* = −0.0005, 95% CI [−0.0007, −0.0004], *t*_3036_ = −6.33, *p* *<* 0.001). Thus, more *Misleading*s were associated with less likelihood of sharing, and the cumulative effect of *Misleading*s in high engagement Tweets was enough to counteract the effect of the *Misleading* to *Like* ratio. If this ratio was all that mattered, the *Misleading* count in Experiment 1 (30% of the *Like* count) would be less effective in reducing sharing than the high *Misleading* count in Experiments 2 and 3 (80% of the *Like* count). However, in an explorative analysis combining the three data sets, we found the *Misleading* count in Experiment 1 to be similarly effective in reducing sharing compared with the high *Misleading* count in Experiments 2 and 3, that is, the interaction term between Experiment and intervention was non-significant (Experiment 1 versus Experiment 2: *B* = −0.08, 95% CI [−0.19, 0.03], *t*_2504_ = 1.30, *p* *=* 0.195; Experiment 1 versus Experiment 3: *B* = −0.05, 95% CI [−0.16, −0.06], *t*_2504_ = 0.850, *p* *=* 0.395). Thus, participants were responsive not only to the *Like* to *Misleading* count ratio but also to the absolute number of *Misleading*s.

## 4. Discussion

We tested the effect of an identity-based misinformation intervention by adding a *Misleading* count next to the *Like* count on social media posts to reduce sharing of partisan misinformation in the US and the UK. Across three experiments, the number of people who reported they would be likely to share the social media posts dropped by 25% in response to the in-group *Misleading* count (versus control) as compared with 5% in response to an adapted version of the accuracy nudge. The *Misleading* count was also more effective when it reflected in-group norms (fellow Democrats/Republicans) compared with the norms of general users and when it was relatively high compared with the *Like* count. The effect of the in-group was not found in the UK sample, which was less politically polarized. Moreover, extreme partisanship, measured as both identity fusion with the political party and identity fusion with leaders, did not undermine the effectiveness of the intervention. These results provide initial evidence that identity-based interventions may be more effective than identity-neutral alternatives for addressing partisan misinformation in polarized contexts.

While completely preventing users from engaging with misinformation—the official Twitter tag condition—was most effective, this strategy relies heavily on moderators and is better suited for unequivocally false rather than misleading content. The *Misleading* count provides an additional and easily scalable layer against misinformation where social media users are able to regulate online content themselves. It had larger cumulative effects (25% fewer people sharing across experiments) than identity-neutral alternatives such as the accuracy nudge (5% fewer people sharing), and it was effective for extreme partisans. The *Misleading* count allows social media users to update their perceived social norms about a given message, which can lead people to conform to these judgements and make behavioural adjustments [25]. In contrast to a *Dislike* count, the *Misleading* count provides normative information on the accuracy of the message. Thus, its function is to convey information about the quality of a message rather than the level of agreement with a given statement.

Using normative cues seems to be more effective in polarized contexts (e.g. US voters compared with UK voters) and for posts on polarizing issues (e.g. immigration) compared with non-polarizing issues (e.g. infrastructure). These findings suggest that polarized contexts offer either greater incentives to conform to in-group norms or greater disincentives not to conform to them—which is consistent with an identity-based approach to misinformation [13,15,16]. As a result, people are more attuned to in-group (versus out-group) norms in highly polarized contexts [38], especially when the issue at stake is fundamental to their status as group members. The effectiveness of in-group norms when group status is most salient (e.g. in polarized contexts and for posts on polarizing issues) also helps clarify why the *Misleading* count was effective even for extreme partisans who reported being fused with either political parties or leaders. Because fused individuals are more driven to match in-group norms [39], the *Misleading* count and other norm-based interventions appear to be particularly compelling for extreme partisans (see also [40]).

Our design offers novel data on the motivations underlying individuals' responses to the *Misleading* count. Partisans could use the in-group norms to identify the most effective posts to promote their views (competitive effectiveness hypothesis). This mechanism could trigger a backfire effect, increasing people's likelihood of sharing posts with a relatively low *Misleading* count compared with the *Like* count, over what would have been expected without any intervention (control condition). However, we did not find support for this hypothesis: even relatively few *Misleading*s (versus *Like*s) reduced participants' likelihood of sharing posts compared with the control condition. Conversely, partisans could use in-group norms as a trusted source to identify true information (civic-mindedness hypothesis). This would involve reductions in sharing in response to any number of *Misleading*s irrespective of *Like*s. In line with this hypothesis, we found an effect of the absolute number of *Misleading*s in reducing participants’ likelihood of sharing posts. Future studies should explore these differences in greater depth.

While the inclusion of a *Misleading* button that people can click on is straightforward to implement and scale, incorporating the in-group condition is more challenging. One option would be to replace the in-group (fellow Democrats/Republicans) with ‘people you follow’ or by AI-generated user subgroups (e.g. ‘people like you’). In this case, the *Misleading* count would only be altered by a particular subgroup, and each user would see a different *Misleading* count, preventing out-group members from abusing the *Misleading* button to ‘attack’ social media posts. This intervention relies on naturally occurring variation in how the in-group evaluates a particular social media post (some will *Like* it, others will tag it as misleading). While it is not clear how many in-group members will be willing to tag specific in-group misinformation as misleading, we found that just a few *Misleading* tags (e.g. 20% of the *Like* count) are enough to have a deterrent effect. Future research should assess if 20% is a realistic assumption and see if people are willing to tag in-group content as misleading. Prior research suggests that crowdsourcing accuracy judgements may be feasible and effective [29].

Similarly to other interventions such as the accuracy nudge [12,13], the *Misleading* count is less effective for conservatives than liberals. This partisan difference could be related to perceptions of the existing norms within these political groups, labelling interventions as punitive and biased [41], or psychological differences in need of closure [42] and accuracy motivation [18]. In the case of the *Misleading* count, this limitation can be partially compensated with a higher total *Misleading* count (e.g. in Experiment 1). Thus, between-group differences in the effect of the intervention notably decrease for high-engagement Tweets with higher total *Misleading* counts.

The main limitation of the present study is that it is a series of controlled experiments with carefully curated social media posts. Although intentions to share are highly correlated with real-world sharing [43], more ecologically valid approaches are necessary to determine its effectiveness in a real-world social media setting. This is especially important for intervention studies that report small to medium effect sizes in samples of panel respondents such as the present study. In a more realistic setting, people would be exposed to a collection of both partisan and nonpartisan social media posts with accurate and inaccurate information and would be able to actually share the posts with others. Related to this, although our *likelihood of sharing* measure is widely employed to assess intentions to share social media posts (e.g. [29]), it could be that it overestimates people's actual likelihood to share posts in the real world. Future research could thus test the proposed intervention within a social media simulation or in field studies.

Moreover, we did not measure how perceived norms about the accuracy of each message changed before and after the intervention. Thus, we cannot directly test whether shifts in perceived social norms mediate the effect of the intervention on sharing intentions. Finally, the current research includes liberal and conservative voters in the US and the UK, limiting the generalizability of the findings to these populations.

## 5. Conclusion

Identity-based interventions that incorporate normative cues appear to be more effective than identity-neutral interventions to counter partisan misinformation among individuals in politically polarized contexts (e.g. US voters). Particularly, pairing partisan misinformation with in-group accuracy judgements reduced misinformation sharing among partisans in the US and the UK. This strategy was effective even for extreme partisans who identified highly with their political leader. Thus, allowing social media users to publicly tag posts as misleading could contribute to stopping the spread of misinformation.

## Data Availability

Supplementary material is available online [44].
